# A Method for Identifying Pollution Sources of Heavy Metals and PAH for a Risk-Based Management of a Mediterranean Harbour

**DOI:** 10.1155/2017/4690715

**Published:** 2017-11-14

**Authors:** Ombretta Paladino, Arianna Moranda, Mahdi Seyedsalehi

**Affiliations:** ^1^Department of Civil, Chemical and Environmental Engineering, Università di Genova, Via Opera Pia 15, 16145 Genova, Italy; ^2^Environmental Chemical Processes Lab, Università di Genova, Savona Campus, Via Magliotto 2, 17100 Savona, Italy; ^3^Department of Environmental Engineering, Science and Research Branch, Islamic Azad University, Tehran, Iran

## Abstract

A procedure for assessing harbour pollution by heavy metals and PAH and the possible sources of contamination is proposed. The procedure is based on a ratio-matching method applied to the results of principal component analysis (PCA), and it allows discrimination between point and nonpoint sources. The approach can be adopted when many sources of pollution can contribute in a very narrow coastal ecosystem, both internal and outside but close to the harbour, and was used to identify the possible point sources of contamination in a Mediterranean Harbour (Port of Vado, Savona, Italy). 235 sediment samples were collected in 81 sampling points during four monitoring campaigns and 28 chemicals were searched for within the collected samples. PCA of total samples allowed the assessment of 8 main possible point sources, while the refining ratio-matching identified 1 sampling point as a possible PAH source, 2 sampling points as Cd point sources, and 3 sampling points as C > 12 point sources. By a map analysis it was possible to assess two internal sources of pollution directly related to terminals activity. The study is the prosecution of a previous work aimed at assessing Savona-Vado Harbour pollution levels and suggested strategies to regulate the harbour activities.

## 1. Introduction

Mediterranean coastal habitats are subject to intense environmental contamination since they are densely populated and characterized by the presence of industrial plants and economic and tourism activities [1, 2]. Both heavy metals (HM) and Polycyclic Aromatic Hydrocarbons (PAH) have been found in sediments of most Mediterranean harbours, including Porto of Vado, Savona, Italy. Savona-Vado Harbour is located in the Ligurian Sea, belonging to the northern part of the western Mediterranean, and the effects of significant urbanization, harbour development and extension, industrial activities, and intensive agriculture and aquaculture, led to a significant coastal pollution [3–5].

The identification of pollution sources in industrial ports is a difficult issue. Contamination of sediments may result in fact both from internal sources of pollution as current and previous accidents during cargo handling and ship loading and from external sources as industrial activities (energy and chemicals production plants, waste water treatment) or intensive agriculture and farming facilities situated near the harbour.

By measuring pollutants concentrations in bottom sediments, some insight can be provided on the sources and sites of accumulation, as well as on the quality of the coastal ecosystem and the potential risk for human beings and marine organisms [6]. Disadvantages in using these data for source identification are in the interpretation of contaminant distributions, which can be made difficult by the different grain size of the collected sediments and their related chemical-physical and transport properties, which can vary even in areas close to each other [7–9].

Moreover, precipitated or adsorbed pollutants can be redistributed by physical processes, and contaminated sediments could be concentrated in depositional areas or transported from harbours to coastal neighboring zones. Advantages in the use of these data are due to the fact that they contain a sort of past memory of all the coastal activities, including unknown accidental events.

Different methods can be used to identify pollution sources by experimental data, depending on the prior knowledge about the location of the possible sources and their possible contribution to pollution. Due to the complexity of the problem a rigorous, physically based, inverse modelling is not usually suggested. Ratio matching [10], zonation [11], and score matching [12] were used to assess point source pollution, while PCA was used for source identification and dimension reduction by Reid and Spencer [13] and Yang et al. [14] and a multivariate statistical approach was proposed for source identification by Li et al. [15].

Alves et al. [16], Loska and Wiechuła [17], and Idris [18] used PCA to assess contamination by heavy metals, Kuo et al. [19] proposed PCA to assess pollution by PAH, and Zitko [20] used this technique to evaluate marine data.

Finally, pattern recognition [21, 22] and cluster analysis [23] were used to group, organize, and reduce the observed environmental dataset, also by coupling it with PCA. Zou et al. [24] also proposed cluster analysis for source identification.

In this paper we propose the use of PCA applied to sampling points (and not to chemicals) in order to individuate the possible sources of internal contamination, followed by a ratio matching of the individuated sampling points in order to cluster the samples supposed to be contaminated by the same internal source.

If the origin of pollution cannot be attributed to point internal sources, some possible external sources of contamination can be individuated.

The procedure is applied to study pollution by heavy metals and Polycyclic Aromatic Hydrocarbons (PAHs) in sediment samples collected in Savona-Vado Harbour (Port of Vado), Italy.

## 2. Materials

### 2.1. Site Description

**Figure 1:**
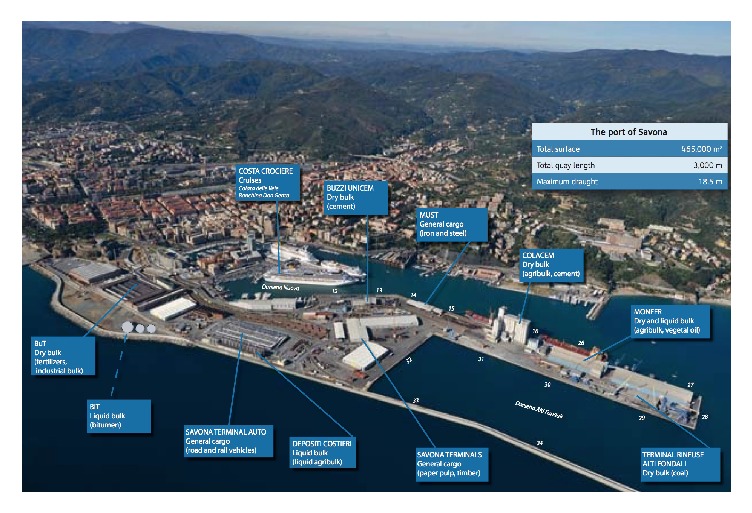
Port of Savona (Savona Port Authority, 2010) [25].

**Figure 2:**
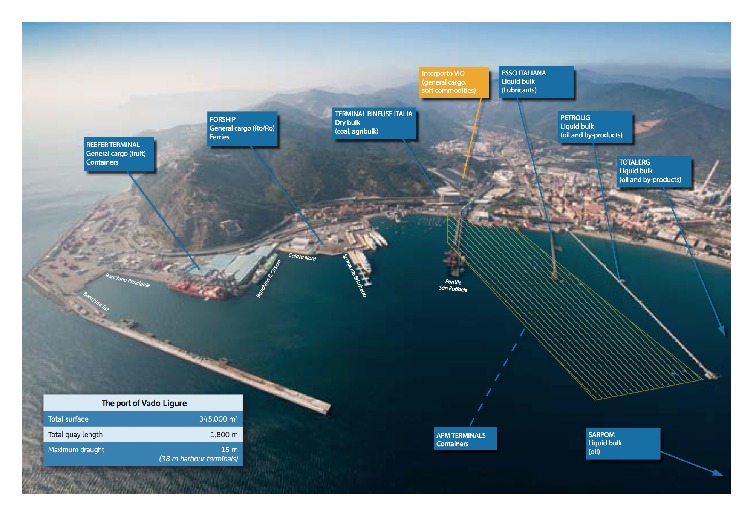
Port of Vado Ligure, 2010 (Savona Port Authority, 2010) [25].

The site under study is Port of Vado, located in Vado Ligure Municipality, Savona, Liguria Region, Italy, northwest. The system, represented by Ports of Genoa, Savona-Vado, and La Spezia, is considered one of the main trade channels for Southern Europe's economy and one of the oldest port systems in the Mediterranean sea, where towns and ports have grown together through thousands of years of history. Port of Savona-Vado is logistically divided into two parts: the former, called “Port of Savona” (Figure 1) is made up of the oldest docks and quays of the ancient port, it is located close to the city (old town) of Savona and was recently renovated and dedicated to leisure boating, cruises and general tourism activities. The latter, farthest from the town and called “Port of Vado” (Figure 2) is located near the industrial area of Vado Ligure and concentrates cargo handling activities.

Identification of pollutant sources in Port of Vado is a complex problem: as for the other two Ligurian Ports, the mountains are very close to the sea, this means that industrial activities, located in the coastal strip near the sea, can contribute to contamination in the port area. Moreover, the rivers are torrential type, and significant gradients are present from the coast to the open sea [26], also contributing to creating correlations among pollutants that may affect the results of a source identification procedure.

Since Vado Ligure is the largest industrial site of the west Liguria, where about 600 small and medium factories and some big plants are located, and its extension is only 24 km^2^ (made of a thin strip of territory that extends from the sea to a kilometer inland), the presence of some possible external sources of contamination should be taken into account when proposing a source identification procedure. Heavy metals contamination of samples collected in Port of Vado could be partially due to a 600 MW thermal power plant that used coal as a fuel (finally closed in 2015); PAH distributions could be affected by one chemical plant that produces and stores petroleum derived additives; finally both heavy metals and PAH could come from three big plants that, respectively, treat and store hydrocarbons, minerals and coal, chemicals, and fertilizers.

Otherwise, possible internal sources of contamination of Port of Vado could be two landing terminals for the drainage of oil-bearing products (Petrolig and TotalErg liquid-bulk) and one terminal for the drainage of lubricants (Esso Italiana liquid-bulk); one landing terminal for discharging anthracite that is also used to embark coke (San Raffaele Dry bulk jetty); the ferry and cargo landing terminal (ForShip RoRo); the west jetty, used for ship refuelling and supplying.

The study area is shown in Figure 2.

### 2.2. Experimental Data

Since container traffic in Port of Vado increased in the last ten years, the Port Authority planned the realization of a new terminal: construction works started in June 2012 and will be completed by the end of 2017. Four complete sampling campaigns were carried out in the area of interest (Figure 2) to assess contamination before and during the construction of the new terminal, according to Sediment Quality Guidelines (SQGs), and to support decisions about dredging-related activities [27]. Sediments were collected in accordance to DL 152/06 of the Italian law, with a box corer (a Van Veen grab sampler was also used in some cases to validate the sampling procedure) and they were mainly constituted by very fine sand/silt. Details about sediment grain size distributions are reported in Paladino et al. [28] and a scheme of the complete dataset is summarized in Table 1.

The first three monitoring campaigns and relative analyses were conducted by Environmental Regional Agency (ARPAL), while the fourth monitoring campaign and relative analyses were conducted by CIMA Foundation and associated laboratories.

The adopted analytical methods were as follows: DM 13/09/99 n. 185 GU 248 (Met II, 1-2-3, Met XI, Met XII) and EPA 3545 and EPA 8270D (heavy metals, HC, and PAH).

**Figure 3:**
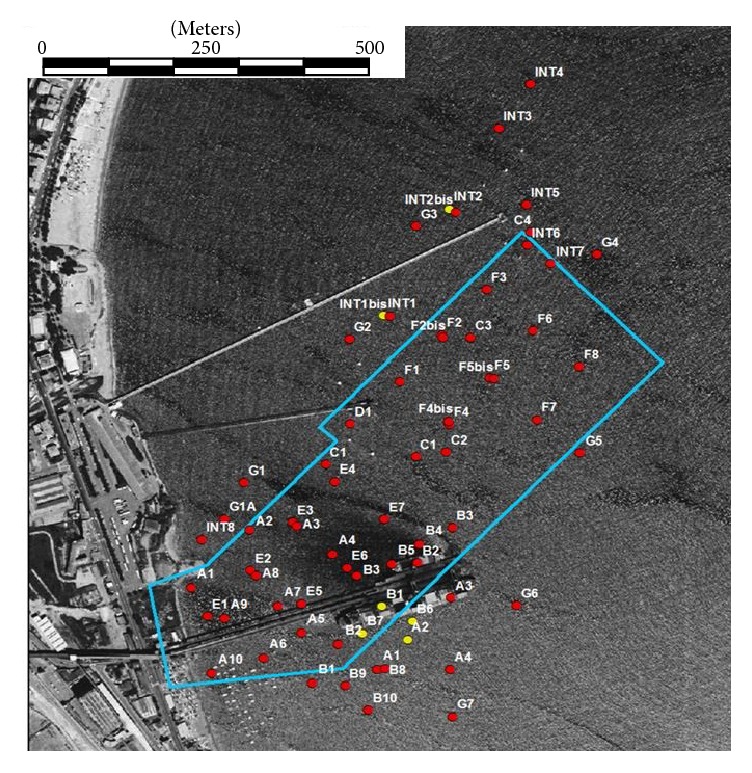
Sampling points (red dots = more than 70% of chemicals exceed limits, yellow dots = less than 70% of chemicals exceed limits, and blue line = new platform).

The position of the total 81 sampling points, as reported in documents produced by the Environmental Regional Agency, can be seen in Figure 3.

Sampling points named A1, A2, A3, A4, B1, B2, and B3, which referred to samples collected in the second campaign, have positions very different from the corresponding sampling points of the first monitoring campaign. We obviously considered them as different sampling points, and a suffix 01 was added to the name of points belonging to the first campaign. Since sampling points *n* F2 and F2bis, F4 and F4bis, and F5 and F5bis are, respectively, very close to each other, we assumed their positions as representative of the same sampling point (F2 = F2bis, F4 = F4bis, and F5 = F5bis) during the identification procedure. Moreover, since in the fourth monitoring campaign no new sampling points were added (samples were collected in points A3, B2, B7, B8, B9, E301, E501, G101, INT701, and INT801, positions already present in the previous campaigns) the total number of independent sampling points (position) considered in the overall study was 68.

## 3. Methods

The novelty of the proposed method consists in the combined use of PCA applied to sampling points (and not to chemicals as in classical procedures aimed at eliminating correlated variables) in order to individuate the possible sources of internal contamination with a ratio matching of the individuated sampling points in order to cluster the samples supposed to be contaminated by the same internal source. If the origin of pollution cannot be attributed to point internal sources, different possible external sources of contamination are individuated by considering the following.

### 3.1. Theoretical Background

#### 3.1.1. Principal Component Analysis

Principal Component Analysis (PCA) is a widely used technique to manage large datasets projecting the original *Z*-dimensional measurement space of *m* variables and *n* samples into a lower *A*-dimensional space. PCA is mainly used for reducing a dataset containing a large number of *m* variables to one space that contains a reduced number of new variables called PCs. The new variables are weighted linear combinations of the original ones and can be used to represent the maximum possible fraction of variability contained in the original dataset without high loss of original information [29–31].

PCA can be carried out in two different ways on the (*m* × *n*) matrix containing the concentration values of *m* chemicals measured in *n* samples of surface sediments: PCA with respect to chemicals and PCA applied to samples.

With the latter method it is possible to identify the outliers, that is, those samples that are not representative of the overall contamination situation. Although the main use of PCA applied to a dataset is the reduction of the *m* variables trying to eliminate correlated variables and samples with less statistical significance, the use proposed for PCA in this paper is the interpretation of the “outliers” in order to individuate some possible spatial patterns in the distribution of variables.

In this work both screen plots and statistics based on the median absolute deviation about the median (MAD) were used to find outliers that could represent pollution sources. The selection of the representative PCs (describing a high fraction of variance) was performed by the screen plot of the eigenvalues [29], while comparison of results was made by evaluating [32–35] how far a point *x* is outlying from the median of the dataset.

A robust measure of this distance *d*(*x*), for one-dimensional datasets *X* = (*X*_1_,…, *X*_*m*_), is given by

(1) $$\begin{array}{c} d\left(\;x\;\right)=\left|\;x-Median\left(\;Xi\;\right)\;\right|\;i=1,m. \end{array}$$

Having calculated the MAD of a dataset, *On*(*x*) represents how many MADs from the median the point *x* is; that is,

(2) $$\begin{array}{c} On\left(\;x\;\right)=\frac{\left|x-Median\left(Xi\right)\right|}{MAD\left(Xi\right)}\;i=1,n, \end{array}$$

and the number *n*, called the outlier cutoff, can be chosen to decide when a point *x* can be defined as an outlier.

#### 3.1.2. Ratio-Matching Technique

Ratio-matching technique is based on the observation that sediment samples whose pollution is due to the same source tend to have similar ratios of trace pollutants concentrations as heavy metals, PCB, and PAH, even if their absolute concentrations in these samples may vary considerably due to dilution and mixing with inert materials as silica or calcite. Comparison of these ratios, evaluated for each individual sample, yields a matrix of similarity coefficients [10], which can be analyzed by a cluster analysis procedure. Sediment samples of similar origin tend to group together in a single cluster, whereas samples in proximity to point source discharges will appear as separate individual clusters. This technique was also used to treat sediment data about harbour contamination in Tranchina et al. [36].

### 3.2. The Proposed Approach

**Figure 4:**
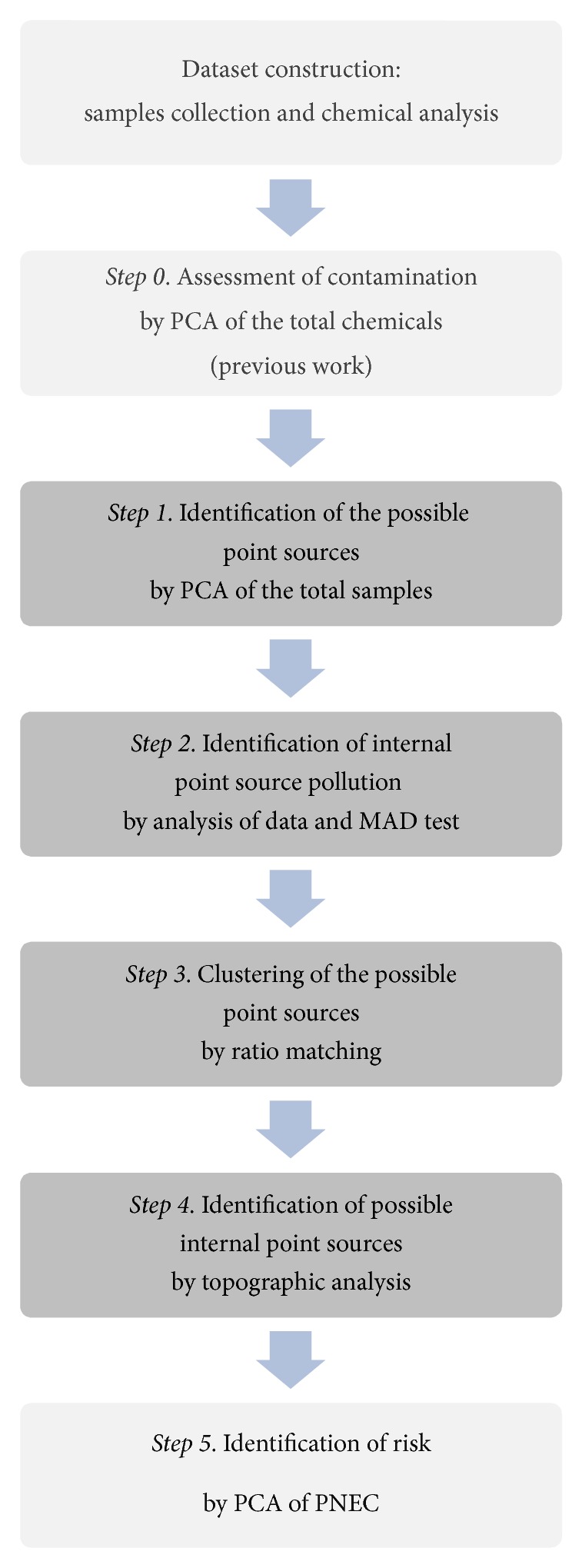
Flow chart of the proposed procedure.

A flow chart of the procedure here adopted (Steps 1–4) is represented in Figure 4.

The procedure should follow the classical PCA on chemicals (Step  0) and is summarized as follows.

**Step 1 (identification of the possible point sources by PCA of the total samples).**

PCA is applied using samples as variables to identify the locations that best describe the pollution in the area. By using the variance-covariance matrix of data, the solution of this problem leads to the elimination of outliers and the individuation of the sampling points more representative of the topography of the chemicals. Since outliers do not represent the total contaminant situation of the area (less variance of the whole dataset), they are representative of a possible localized source of internal pollution.

**Step 2 (identification of internal point source pollution).**

Identification of chemicals that better describe the pollution of each outlier (possible point source): since an outlier is a sampling point not representative of the site pollution, chemicals that better describe the pollution of each outlier are to be identified. This is made by analyzing the matrix of data for each outlier and by calculating *On*(*x*) by (1).

**Step 3 (clustering of the possible point sources).**

Ratio matching is applied by construction of the matrix of similarity coefficients as suggested in [11]. In case of *n* samples and *m* chemicals, for each sample the concentration value of each chemical parameter is divided by concentration values of each of the other chemicals, resulting in (*m* × *m*) matrix for each sample. Only the upper triangular part is considered for a total of *n* upper unitriangular matrixes. Comparison of two generic samples *i* and *j* is made by dividing each element of the matrix *i* by the corresponding elements of the matrix *j*. Clustering can be made by considering the total behaviour of PAH or heavy metals contamination or a particular subset of PAH or heavy metals correlated to the hypothesized point sources.

**Step 4 (identification of possible internal point sources).**

By using a map of the harbour (Figure 1) the terminals or ship routes closer to the clusters are individuated. A check about the materials moved by these terminals suggests confirming or not confirming the contribution to pollution by the assessed clusters.

An additional but optional Step  5 can be carried out in order to assess environmental risk. A PCA by using the variance-covariance matrix of sampling points and contamination levels expressed as ratio between concentration and the Predicted No-Effect Concentration (PNEC) is used to assess point sources at the highest risk.

The procedure was applied to the whole dataset. Sampling points of the fourth campaign have been chosen on the basis of the results of the first step of the proposed procedure and in order to eliminate possible experimental gross-errors. Moreover, the number of PAHs searched for in the fourth campaign was increased in order to obtain more information about some possible sources of contamination.

## 4. Results and Discussion

### 4.1. Step  0: PCA of the Total Chemicals *m*

As discussed in Section 3.1 the proposed procedure for assessing the possible sources of contamination can be carried out when PCA on chemicals is completed. PCA was previously applied to the variance-covariance matrix of the concentration values measured in all of the collected sediments, expressed as hazard quotients (HQ) with reference to APAT-ICRAM/2006 LCL limits or DL 152/06 limits.

The complete analysis for the first three monitoring campaigns and relative results are reported in the paper of Paladino et al. [28]. Main results of this PCA, in terms of weights of chemicals on the overall variance, are shown in Table 1 (column A) with Zn, Pb, H-C (C > 12), Sum-PAH, Cu, Hg, pyrene, Cd, As, Sn, and benzo-anthracene being identified as the most important pollutants. Since a high percentage of the overall variance was represented by the first PC (about 99%) with the weight of Zn equal to about 77% of the overall variance, pollution by Zn was interpreted as reflecting the typical local nonpoint pollution of the area [37], while the second PC was considered to describe random variations departing from the typical situation of pollution, as suggested by Jolliffe [29]. So, in order to find the most important chemicals, PCA was carried out again after distributed contamination of Zn was eliminated. With the new PCA the first eigenvalue represented about 85% of the overall variance, the second one represented the 13%, and the third one the 1% and results are shown in Table 2 (column B).

### 4.2.  Step 1: PCA of the Total Samples *n*

**Figure 5:**
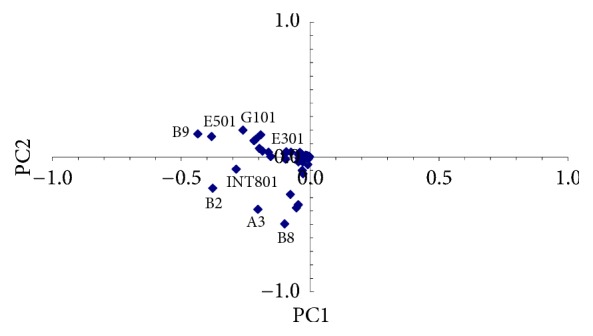
PCA of the total samples and possible point sources.

PCA was applied to the variance-covariance matrix of the *n* position of the samples, considered as main variables. To identify the outliers with the screen plot method a plot of scores in the coordinates of the first two principal components PC1 (99% of variance) and PC2 (0.5% of variance) was used (Figure 5). In this analysis, as discussed in Section 3.1, the outliers can be interpreted as possible internal sources of pollution in the area. The most important point sources are identified: B9, E501, B2, INT801, A3, B8, and G101.

In order to verify if PCA on total samples can be sensitive to errors, sampling points of the fourth campaign have been chosen on the basis of the results of this step: 28 chemicals were searched for within 10 sampling points, of which B9, E501, B2, INT801, A3, B8, G101, and E301 were individuated by PCA (Figure 5), plus INT701 and B7, and 5 replicas for the three sampling points B2, INT801, and E301 for a total of 22 samples.

Moreover, the number of PAHs searched for in the fourth campaign was increased in order to obtain more information about some possible sources of contamination described by samples INT801, B2, and E301, showing high deviation levels (less variance of the whole dataset) for chrysene, indenopyrene, and pyrene.

Step 1 was repeated by including data from the fourth monitoring campaign and all the outliers were confirmed.

**Figure 6:**
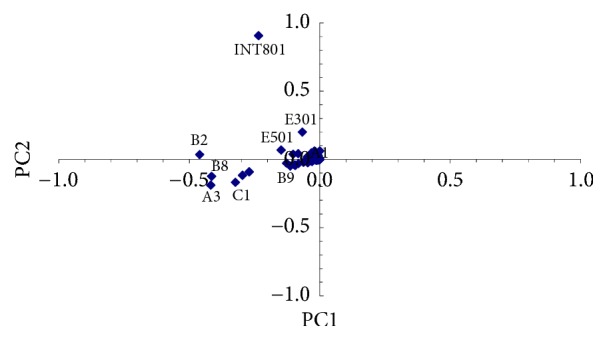
PCA of the total samples and possible PAH/H-C point sources.

**Figure 7:**
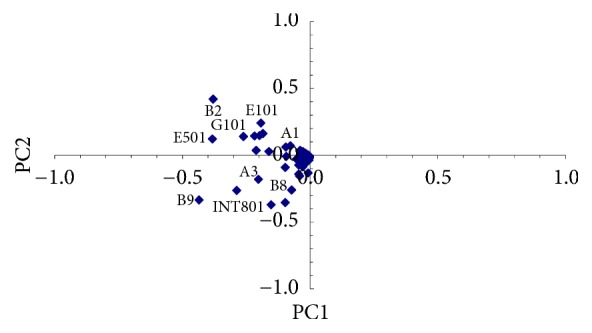
PCA of the total samples and possible heavy metals point sources.

PCA of the total samples was also separately carried out for PAH/H-C and metals. Results are shown in Figures 6 and 7, respectively.

By observing Figure 6 it can be noticed that PC1 is mainly represented by sampling point INT801, showing high deviation levels for Sum-PAH while PC2 is mainly represented by sampling point B2, showing high deviation levels for hydrocarbons with C > 12, followed by sampling points A3 and B8.

With reference to Figure 7, PC1 is almost similarly represented by sampling points B2, B8, and INT801, showing some appreciable but not very high deviation levels for Zn. PC2 is mainly represented by sampling points B9 and E501, both showing high deviation levels of Cd.

### 4.3.  Step 2: Identification of the Internal Point Source Pollution

To conduct source identification we examined outliers by calculating *O*_*n*_ for all of the sampling points and chemicals. For a chosen value of *O*_*n*_ considered as the inferior limit, precise identification of the possible chemical source is possible. Results obtained for *O*_*n*_ = 4 gave the following resulting ordered list of possible point sources: B9, B2, INT801, E501, and A3. For *O*_*n*_ = 5 the list was as follows: INT801, B9, E501, B2, A3, B8, and E301.

By using this approach, the possible pollution sources in the area appear in a different order than that shown in Figure 5 but well matched those found with the screen plot approach, indicating that the procedure based on PCA is definitely robust. By varying *O*_*n*_ or the chosen limit to consider an outlier with the screen plot approach, the number of possible internal sources to be considered can be modified.

Results confirm sampling point INT08 as an outlier for contamination by chrysene, indenopyrene, and Sum-PAH, in good agreement with the screen plot procedure (Figures 5 and 6, where Sum-PAH was the pollutant with higher deviation). Results also confirm B2, A3, and B8 as possible representative point sources of hydrocarbons with C > 12, in agreement with the screen plot procedure (PC2 in Figure 6). Outliers B9 and E501 were confirmed as possible point sources of Cd, in agreement with the screen plot procedure (reported in Figure 7, where Cd was represented by PC2). Finally, results obtained by calculating *O*_*n*_ = 5 do not confirm sampling points B2, B8, and INT801 as possible point sources of Zn, as suggested by the screen plot procedure (PC1 in Figure 7) but confirm them as previously observed after performing Step  0, a nonpoint pollution by Zn.

### 4.4.  Step 3: Clustering of Possible Point Sources

68 upper unitriangular matrixes (28 × 28) were computed and compared. A confidence interval of 10% was defined to approve ratio-matching results. This means that ratio values (between similarity coefficients) in the interval 0.90–1,10 are considered valid to cluster samples. Three interesting clusters were individuated: the first could be associated with contamination by heavy metals (Cd, Pb, and Hg) and is related to samples A3, B6, B8, and B9. The other two clusters are related to samples B2, B8, and B9 (H-C with C > 12 and PAH) and INT1, INT1bis, INT2, INT2bis, INT3, and INT4 (mainly PAH).

Sampling points INT801, E301, and E501 resulted as single clusters.

### 4.5.  Step 4: Identification of Possible Internal Sources

By using a map of the harbour (Figure 2) the terminals and ship routes closer to the clusters were individuated. A check about the materials moved by these terminals (documents on cargo handling) suggested confirming the contribution to pollution by some individuated clusters. In particular, results may suggest liquid-bulk terminal as a probable internal point source for PAH pollution. Since the other two clusters are both situated in the surrounding area to the south of the dry bulk called “terminal rinfuse Italia,” pollution by heavy metals and H-C is probably due to the terminal activity, since this terminal is used for discharging anthracite and to embark coke. Finally, clusters described by single sampling points INT801, E301, and E501 do not seem correlated to continuous internal activity of the harbour, but probably they refer to recent accidental spills during handling or, as the case of point source INT801, to the external activity represented by the shipyard located on the coast very close to the sampling point and probably using cleaning solvents. In order to complete the source identification procedure, the contribution to pollution of the most important industrial sites near the harbour should be investigated. Since a possible source of heavy metals contamination was individuated in the 600 MW thermal power plant using coal, but the plant was stopped in 2015, the work focused only on internal point pollution sources.

### 4.6. Step  5: Identification of Possible Risk

Once point source pollution by heavy metals and PAH was assessed, a final step was carried out in order to identify possible environmental risk. PCA was used, using the variance-covariance matrix of sampling points and PNEC thresholds. Some different methods are available to evaluate PNEC for sediments [38]; among all, the method here used was based on aquatic toxicity data and on the Assessment Factors (AF) approach when only acute sediment toxicity data are available. In case of missing data we used the following formula:

(3) $$\begin{array}{c} {PNEC}_{sediment}=K_{p}*{PNEC}_{water}, \end{array}$$

where *K*_*p*_ [l/Kg] is the partition coefficient between sediment and water.

**Figure 8:**
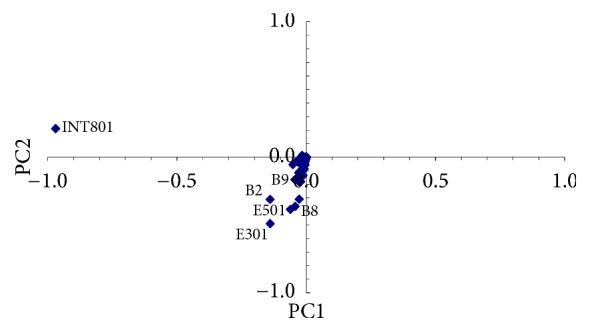
PCA of the total samples by PNEC and sampling points at the highest environmental risk.

Results are shown in Figure 8, where sampling points E301, B2, E501, B8, and INT801 are at the highest environmental risk.

**Figure 9:**
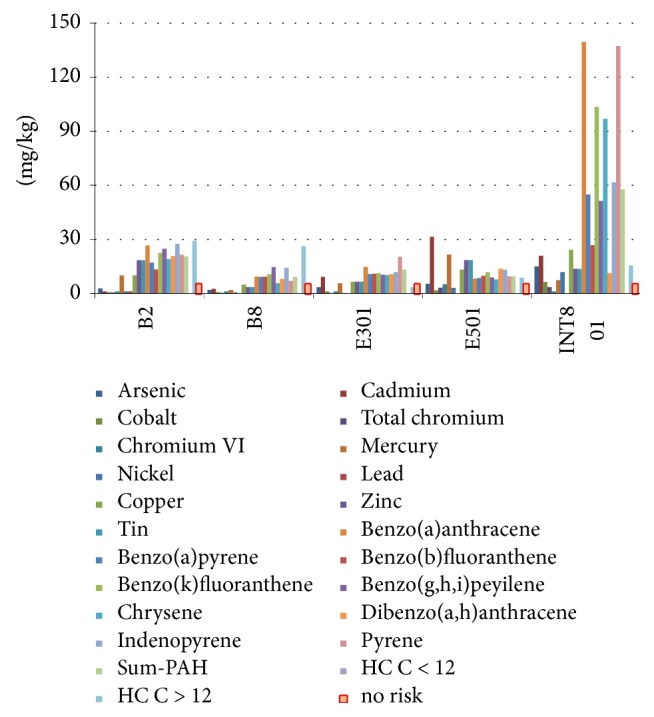
MAD test for sampling points at highest environmental risk (all sampling campaigns).

Finally, Figure 9 shows results (*O*_*n*_ = 5) for MAD test of the five sampling points individuated on the basis of PNEC: INT801, E301, B2, E501, and B8.

By observing Figures 8 and 9 we can state that points B2 and B8 are at high environmental risk. Since from the previous steps of the proposed procedure their pollution could be due to “terminal rinfuse,” a risk-based management approach should provide a deep control and possibly a change in the ship discharging and loading procedures carried out at this terminal. Moreover, pollution of points INT801, E501, and E301 seems to be linked to single accidents during cargo handling. In this case the risk-based management approach must improve the general safety procedures in order to reduce accidents.

## 5. Conclusions

A procedure to cluster and to assess internal sources of sediment contamination in Savona-Vado Harbour was proposed, applied, and discussed. The procedure was based on PCA of total samples followed by ratio-matching and was divided into five steps.

Techniques based on PCA are often used to manage large sets of contamination data. The main goals reached by using these techniques are the extraction of information about the chemicals involved and reduction of the dataset. In this work PCA was employed to obtain useful information on point source pollution. The first step of the procedure allowed the assessment of 8 main possible point sources. A refining second step allowed surely classifying 1 sampling point as a possible PAH source, 3 sampling points as C > 12 point sources, and 2 sampling points as Cd point sources. Clustering by ratio-matching performed at Step 3 allowed grouping some sampling points as probably contaminated by the same point source. By performing an analysis of the harbour area and relative internal activities, it was possible to assess two internal sources of pollution: the former is related to the liquid-bulk terminal activity (oil and by-products, lubricants) and could be considered a potential PAH point source; the latter is related to the dry-bulk terminal activity and in particular to anthracite and coke handling and it could be considered potential Cd, Pb, Hg, and H-C > 12 point source. Three single point clusters cannot be attributed to continuous harbour activities, and probably they could be related to accidental spills.

Finally application of PCA on sampling points dataset by using PNEC thresholds allowed finding the 5 sampling points at the highest environmental risk.

## Figures and Tables

**Table 1:** Scheme of the monitoring campaigns.

	1st campaign: 04/2006	2nd campaign: 05–07/2008	3rd campaign: 02/2009	4th campaign: 01/2010
Collected samples	83	103	27	22
Sampling points	24	37	10	10
Searched heavy metals	11	11	11	11
Searched PAH	10 + 1	10 + 1	10 + 1	14 + 1
Searched HC	2	2	2	2

**Table 2:** Weights of each chemical on the overall variance.

PCA of chemicals	(A) % overall variance	(B) % overall variance (after Zn elimination)
Arsenic	0.31	1.23
Cadmium	0.36	1.48
Cobalt	0.01	0.10
Total chromium	0.09	0.45
Chromium VI	0.00	0.00
Mercury	0.48	1.75
Nickel	0.15	0.67
Lead	13.75	51.72
Copper	2.09	7.80
Tin	0.14	0.91
Zinc	76.29	—
Benzo(a)anthracene	0.15	0.73
Benzo(a)pyrene	0.10	0.47
Benzo(b)fluoranthene	0.10	0.43
Benzo(k)fluoranthene	0.09	0.40
Benzo(g,h,i)perylene	0.07	0.30
Chrysene	0.15	0.70
Dibenzo(a,h)anthracene	0.02	0.06
Indeno(1,2,3-c,d)pyrene	0.06	0.27
Pyrene	0.31	1.43
Sum-PAH	0.86	3.89
Hydrocarbons C < 12	0.00	0.01
Hydrocarbons C > 12	4.39	25.19
